# A case report: Gliosarcoma associated with a germline heterozygous mutation in *MSH2*

**DOI:** 10.3389/fneur.2024.1388263

**Published:** 2024-05-09

**Authors:** Yuhan Wang, Zhiyuan Zhang

**Affiliations:** ^1^Medical School, Nanjing University, Nanjing, China; ^2^Department of Neurosurgery, Jinling Hospital, Affiliated Hospital of Medical School, Nanjing University, Nanjing, China

**Keywords:** case report, germline heterozygous mutation, gliosarcoma, Lynch syndrome, *MSH2*

## Abstract

Gliosarcoma is a rare subtype of glioblastoma (GBM) with a shorter medical history and a worse prognosis compared to other Grade 4 gliomas. Most gliosarcomas are sporadic, but it is undeniable that a small percentage are linked to germline mutations and several inherited cancer susceptibility syndromes, including Lynch Syndrome (LS). The authors present a case of a primary mismatch repair-deficient gliosarcoma in LS. A 54-year-old Chinese male patient was admitted to the hospital with a history of facial asymmetry for over 1 month and right temporo-occipital pain for 5 days. Head MRI revealed a complex mass lesion in the right frontoparietal region, consisting of cystic and solid components. The patient’s history of colon malignancy and family history of rectal carcinoma were noteworthy. Postoperative pathology indicated the presence of gliosarcoma with high-frequency microsatellite instability (MSI-H) and mismatch repair deficiency (MMRD). Further genetic testing results confirmed a germline heterozygous mutation in *MSH2*, which is considered the gold standard for diagnosing LS. This case report enriches the existing literature on germline *MSH2* mutations and gliosarcomas. It highlights the importance for neurosurgeons to consider possible hereditary disorders when treating patients with a history of concurrent tumors outside the nervous system. Genetic testing is crucial for further identification of such disorders.

## 1 Introduction

Gliosarcoma is a rare primary central nervous system malignancy classified as a variant of glioblastoma (GBM) in the 2021 WHO Classification of Tumors of the Central Nervous System, accounting for 2–8% of all GBMs (1, 2). Gliosarcoma exhibits histologic features of both glioblastoma and soft tissue sarcoma, with a shorter median survival and poorer prognosis compared to other GBMs in general (2, 3).

Brain tumors, including gliosarcomas, are mostly sporadic, and only a small percentage of them are associated with hereditary cancer susceptibility syndromes, such as Lynch syndrome (LS) (4, 5). LS is an autosomal dominant tumor syndrome with a prevalence of approximately 3–5% of all bowel cancers. It can increase the risk of developing tumors in the colorectum and other body parts, including the endometrium, ovaries, gastrointestinal tract, liver, gallbladder, upper urethra, brain, skin, and so on (6, 7). Germline heterozygous mutations in mismatch repair (MMR) genes, such as *MLH1, MSH2, MSH6,* and *PMS2*, underlie LS predisposition. Rarely, mutations in *EPCAM* may result in high methylation of the promoters of *MSH2*, causing epigenetic silencing of a structurally intact *MSH2* (6, 8).

Several studies have indicated that the risk of primary brain tumors, particularly high-grade gliomas, increases by approximately four times in individuals with LS (4). However, there are currently few clinical cases of LS patients with gliosarcoma available (4). Here we report a case of a patient with gliosarcoma, which was ultimately diagnosed as LS through a comprehensive analysis of the patient’s past medical and family history, as well as histopathological and molecular test results.

## 2 Case report

**Figure 1:**
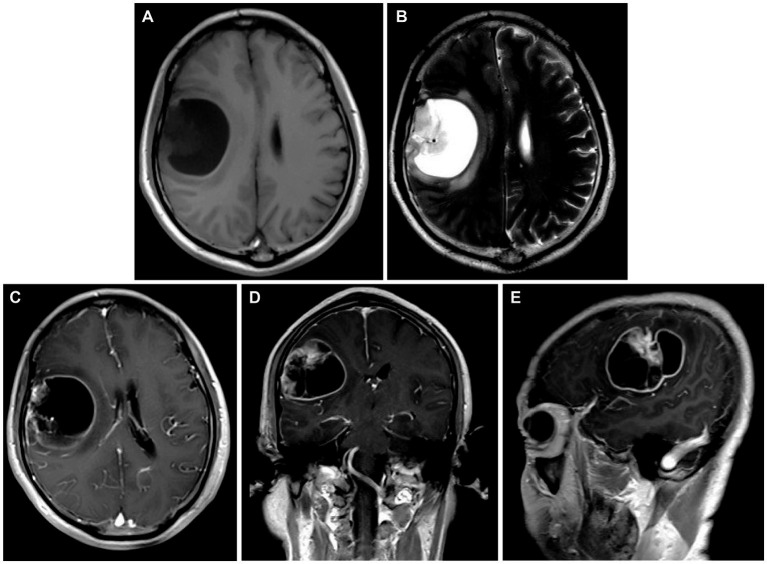
**(A,B)** Preoperative head MRI T1-weighted sequences and T2-weighted sequences demonstrating a rounded-like cystic solid lesion in the right frontoparietal lobe. **(C–E)** Axial, coronal, and parasagittal MRI in T1-weighted contrast-enhanced sequences showing the markedly enhanced margins and solid components of the lesion.

A 54-year-old Chinese male patient was admitted to the Department of Neurosurgery, Jinling Hospital (Nanjing, Jiangsu Province, China) on October 20, 2023. He presented with a skewed left side of the mouth for over 1 month, along with right temporo-occipital pain lasting for 5 days. Before admission, he underwent head CT and MRI scans that showed a right temporoparietal lobe mass at several different hospitals. The admission physical examination revealed his overall good condition, with only minor signs of damage to the facial and hypoglossal nerves. The head MRI showed a rounded-like cystic solid lesion in the right frontoparietal lobe, measuring 53 × 44-mm, with hyperintensity surrounding it on T2-weighted images. Contrast-enhanced scans revealed significant enhancement of the margins and solid components of the lesion (Figures 1A–E). The past medical history of the patient was noteworthy. In 2001, the patient underwent surgery for a transverse colon tumor. Fourteen years later, a malignant tumor with colonic polyps reappeared in the ileocecal region, and he underwent standard chemotherapy treatment after a right hemicolectomy. Unfortunately, he underwent a Dixon procedure and received postoperative chemotherapy after being re-examined for rectal cancer during a follow-up in 2019. Moreover, his mother passed away from rectal malignancy, but the patient did not provide many details about it. His past medical and family history closely matched the familial diagnostic criteria for LS. Regrettably, the LS screening tests were not conducted during the previous visits, so any significant histological and genetic findings from those previous resections were not available.

**Figure 2:**
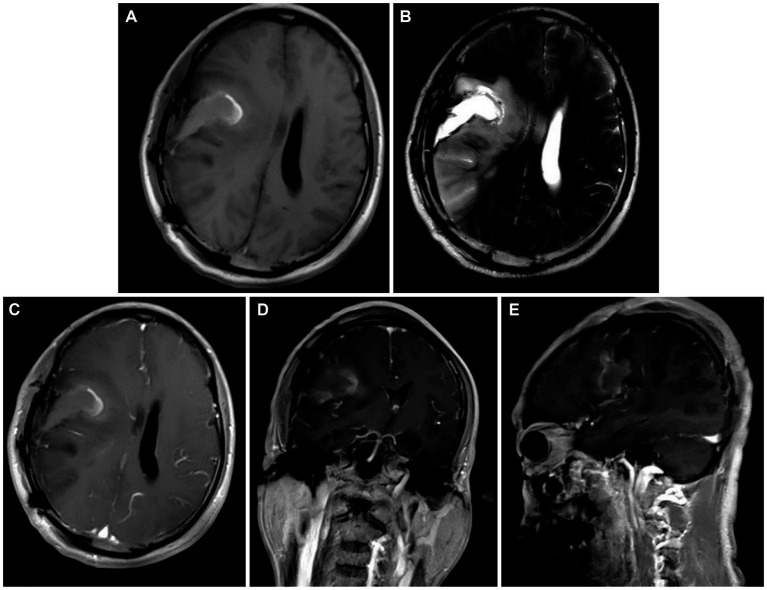
**(A,B)** Postoperative head MRI T1-weighted sequences and T2-weighted sequences illustrating complete resection of the cystic solid lesion in the right frontoparietal lobe. **(C–E)** Axial, coronal, and parasagittal MRI in T1-weighted contrast-enhanced sequences showing no significant enhancement in the surgical area.

**Figure 3:**
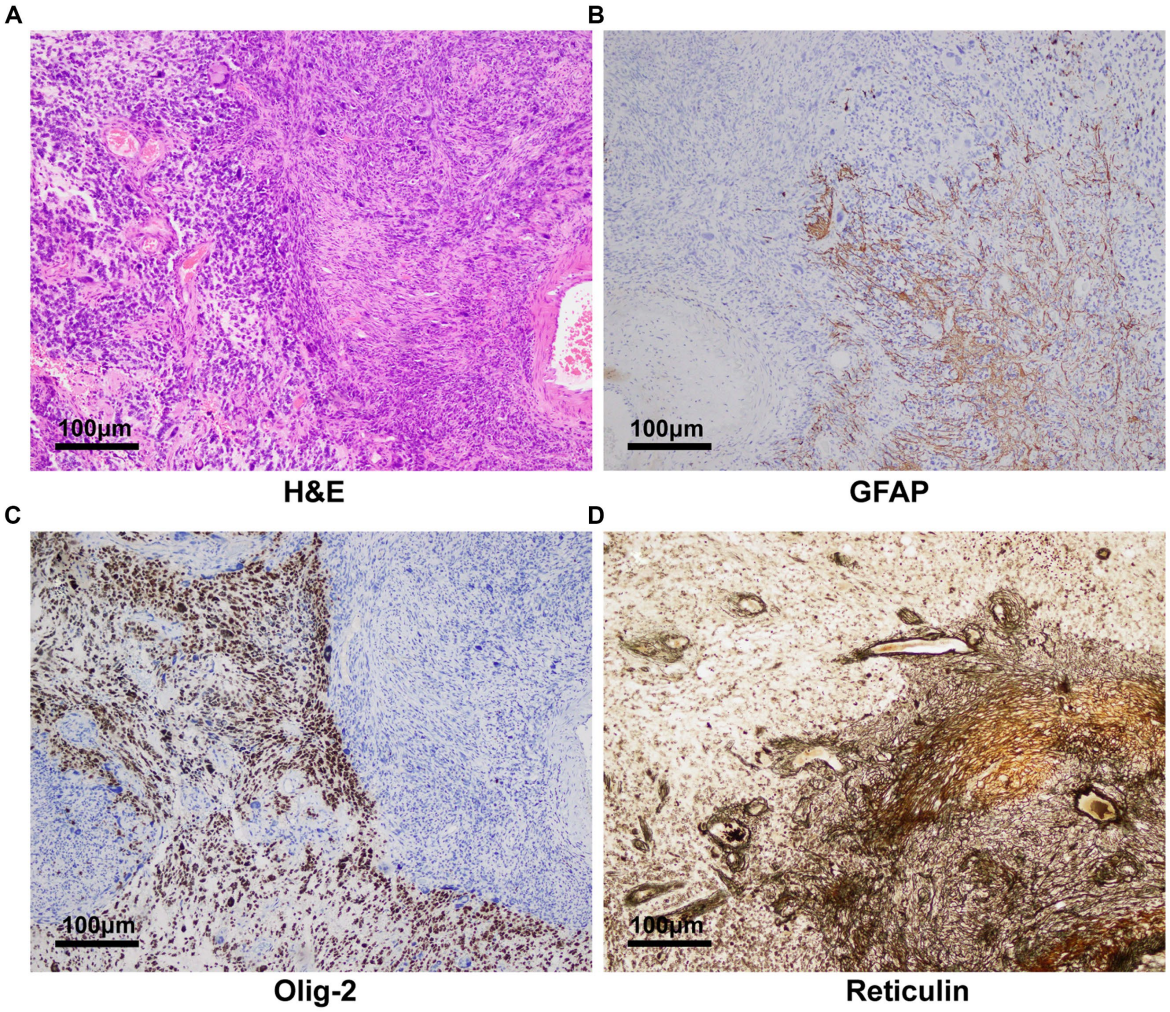
Pathology and immunohistochemistry revealing glioblastoma with a small focal area of gliosarcoma. **(A)** H&E staining (100×) showed the tumor was mixed by glioma and sarcoma components. **(B)** Glial fibrillary acidic protein and **(C)** olig-2 was immunohistochemically positive in the tumor (100×). **(D)** Reticulin stains (100) disclosed abundant reticular fibers within tumor cells in the sarcomatous field.

We thoroughly informed the patient and his family about the necessity of the surgery, its risks, and potential follow-up medical procedures, and ultimately obtained written consent. A right frontotemporoparietal craniotomy was performed as scheduled. After incising the dura mater, a grayish-red mass was observed in the right frontotemporoparietal lobe, displaying abundant blood supply. After releasing the yellow-green cystic fluid, the tumor was dissected along the boundaries of its capsule wall under the operating microscope. Postoperative contrast-enhanced MRI showed gross total resection was achieved (Figures 2A–E). The final pathology results confirmed a high-grade glioma with extensive necrosis (Figure 3A). Immunohistochemistry for glial fibrillary acidic protein (GFAP) and olig-2 was positive (Figures 3B,C), and reticulin stains revealed abundant reticular fibers within the tumor (Figure 3D). Combined with the aforementioned immunohistochemical results and molecular testing, the patient was diagnosed with glioblastoma (*IDH* wild-type, WHO grade 4) with a small focal area of gliosarcoma. The patient’s past medical history of three primary colonic malignancies and the family history of cancer prompted a comprehensive investigation into possibly potential genetic syndromes, particularly for LS. The DNA of the gliosarcoma biopsy specimen was evaluated for microsatellite instability (MSI) using a PCR-based technique, and exhibited high-frequency MSI (MSI-H) and mismatch repair deficiency (MMRD). Genetic testing results confirmed a pathogenic heterozygous germline mutation in *MSH2* (exon 4 c.765_766del p. A256Cfs*27) and a somatic mutation in *MSH6* in this patient. The patient received postoperative symptomatic treatments, including dehydration, and recovered well with a normal functional outcome. After discharge, the patient received the STUPP regimen, which required him to undergo regular local radiotherapy (60 Gy/30 fx) and concurrent chemotherapy with temozolomide (TMZ) (75 mg/m^2^ per day), along with six cycles of TMZ maintenance therapy. The patient is making a good recovery with no significant adverse effects.

## 3 Discussion

In this case report, we present the comprehensive diagnostic considerations and treatments for a 54-year-old Chinese male patient diagnosed as gliosarcoma with a germline heterozygous mutation in *MSH2.* This case highlights the importance of genetic screening in certain instances of brain tumors, especially in patients with a past medical and family history of tumors.

Most of brain tumors, including gliosarcoma, are typically non-hereditary and are primarily influenced by somatic gene mutations and environmental factors. In this case, we found frequent mutations in *TERT*, *PTEN*, *TP53*, and *NF1* in gliosarcoma, which are associated with well-known cellular pathways such as cell cycle regulation, genetic stability, and cellular proliferation (9). Consistently, genetic analysis by Zaki et al. (10) revealed that the most frequently mutated genes in gliosarcoma are *TERT* promoters (92%), *PTEN* (66%), *TP53* (60%), and *NF1* (41%), reflecting the complex and diverse pathogenesis of gliosarcoma.

Nevertheless, in a few cases, the occurrence of primary brain tumors is probably related to hereditary cancer susceptibility syndromes, such as LS, despite the low incidence (4, 11–13). The accurate progression of DNA replication depends on the MMR system, which plays a significant role in recognizing and repairing DNA mismatches. This process is regulated by the *MLH1, MSH2, MSH6, PMS2*, and *EPCAM* genes. LS is caused by MMRD, which leads to an accumulation of point mutations and MSI, increasing susceptibility to multiple cancers (14). In general, MSI-H is rare in sporadic brain tumors. However, current researches have shown that GBMs associated with LS almost entirely exhibit MSI-H (4), which is consistent with this case.

It has been reported that LS carriers have a cumulative lifetime risk of 70% for colorectal cancer at age 70 years, 27–71% for endometrial cancer, and 12% for urinary tract cancer, with an additional incremental risk of cancers of the pancreas, brain, small bowel, and skin (15). Although brain tumors have been considered uncommon in patients with LS, LS indeed increases the risk of brain tumors 4-fold, especially high-grade gliomas (4), and individuals with LS have an approximately 3% risk of developing brain tumors in a lifetime (16). A study from Denmark revealed that primary brain tumors developed in 41 out of 288 LS families, with the main causative mutations occurring in *MSH2*. Among these tumors, GBMs were the predominant histologic subtype (56%), followed by astrocytomas (22%) and oligodendrogliomas (9%) (17).

The genetic basis of LS resembles that of constitutional mismatch repair deficiency syndrome, with the former being heterozygous and the latter being homozygous or compound heterozygous mutations in MMR genes. The distribution of germline mutations in LS brain tumors predominantly occurs in *MLH1* (15%) and *MSH2* (68%), while mutations in other genes account for a smaller percentage (5, 17, 18). Furthermore, it has been proven that there is a close correlation between cancer risk and the affected MMR genes (17, 19). Individuals with pathogenic mutations in *MSH2* have a significantly higher incidence of primary brain cancers, with a risk of 2.5%, compared to 0.5 and 0.8% with *MLH1* and *MSH6* mutations, respectively (17, 20). Nonetheless, there is only an incidence of 0.88% of developing brain tumors in LS patients with *MSH2* or *MLH1* mutations (5, 21).

In terms of treatment, there is currently no cure for LS. Surgical resection of the lesion followed by targeted chemotherapy and immunotherapy is the main treatment approach. Clinically, the gold standard for postoperative treatment of newly diagnosed GBMs is the STUPP regimen (22). A study demonstrated that these treatments were found to have a substantial impact on median survival, increasing it from 12.1 months (radiotherapy only) to 14.6 months, and boosting the two-year survival rate of patients from 10.4 to 26.5% (22). However, this study did not differentiate between sporadic or genetically related GBMs cases. Actually, the efficacy of TMZ can be influenced by factors such as O(6)-Methylguanine DNA methyltransferase (MGMT) and MMR (23). MGMT is a DNA repair enzyme that reverses DNA damage caused by alkylating agents, rendering tumors insensitive to TMZ chemotherapy (24). In GBMs, the MGMT promoter regions are often methylated to some extent, rendering this group of individuals more sensitive to TMZ (25). However, the genetic testing results of this patient showed that the MGMT promoter methylation status was negative. Therefore, his clinical benefit with TMZ chemotherapy would be less than that of positive patients. We suggested tumor treating fields therapy as a complementary treatment to the patient, but he refused.

A limitation of this case is the lack of long-term follow-up data on the patient’s response to concurrent radiotherapy and chemotherapy due to the short duration. We will continue monitoring the patient in the long run to assess his health.

A study suggested that genetic screening for MMRD and LS should be systematic in GBMs with wild-type *IDH*, diagnosed under the age of 50 years (13). Accurate identification of at-risk populations will assist in developing early disease surveillance and intervention programs for detecting precancerous lesions or early-stage tumors. Studies have shown that the early stages of LS-related tumors exhibit greater sensitivity to immune checkpoint inhibitors (26), and patients with GBMs resulting from underlying LS may benefit from these medicines like pembrolizumab (27). Therefore, keenly identifying patients with suspected LS, confirming the diagnosis with genetic testing, and subsequently developing individualized treatment plans for them are of great significance for improving patients’ quality of life in the future.

## 4 Conclusion

In conclusion, this case highlights that underlying inherited cancer susceptibility syndromes are significant considerations when dealing with patients with a history of concurrent tumors outside the nervous system. Patients’ relevant histories, including past medical history and family history, can provide valuable information for identification. Furthermore, identifying potential germline mutations will contribute to the selection of treatment options for patients and will have implications for future cancer surveillance for both the patients and their family members.

## Data availability statement

The original contributions presented in the study are included in the article/supplementary material, further inquiries can be directed to the corresponding author.

## Ethics statement

The studies involving humans were approved by the Ethics Committee of Jinling Hospital, affiliated Hospital of Medical School, Nanjing University. The studies were conducted in accordance with the local legislation and institutional requirements. The human samples used in this study were acquired from brain tumor samples taken during surgery. Written informed consent for participation was not required from the participants or the participants’ legal guardians/next of kin in accordance with the national legislation and institutional requirements. Written informed consent was obtained from the individual(s) for the publication of any potentially identifiable images or data included in this article.

## Author contributions

YW: Writing – original draft. ZZ: Funding acquisition, Writing – review & editing.
